# Multivariate analysis of the Fugl-Meyer outcome measures assessing the effectiveness of GENTLE/S robot-mediated stroke therapy

**DOI:** 10.1186/1743-0003-4-4

**Published:** 2007-02-19

**Authors:** Farshid Amirabdollahian, Rui Loureiro, Elizabeth Gradwell, Christine Collin, William Harwin, Garth Johnson

**Affiliations:** 1 Think Lab, The University of Salford, Maxwell Building, Salford, M5 4WT, UK; 2 Department of Cybernetics, University of Reading, Reading, RG6 6AY, UK; 3 Community Therapy Team Florence Desmond Day Hospital, Royal Surrey County Hospital, Guildford, Surrey, GU2 7XX, UK; 4 Department of Neurorehabilitation, South Block Annexe, Royal Berkshire Hospital, London Road, Reading, RG1 5AN, UK; 5 Centre for Rehabilitation and Engineering Studies, School of Mechanical and Systems Engineering, University of Newcastle upon Tyne, Newcastle, NE1 7RU, UK

## Abstract

**Background:**

Robot-mediated therapies offer entirely new approaches to neurorehabilitation. In this paper we present the results obtained from trialling the GENTLE/S neurorehabilitation system assessed using the upper limb section of the Fugl-Meyer (FM) outcome measure.

**Methods:**

We demonstrate the design of our clinical trial and its results analysed using a novel statistical approach based on a multivariate analytical model. This paper provides the rational for using multivariate models in robot-mediated clinical trials and draws conclusions from the clinical data gathered during the GENTLE/S study.

**Results:**

The FM outcome measures recorded during the baseline (8 sessions), robot-mediated therapy (9 sessions) and sling-suspension (9 sessions) was analysed using a multiple regression model. The results indicate positive but modest recovery trends favouring both interventions used in GENTLE/S clinical trial. The modest recovery shown occurred at a time late after stroke when changes are not clinically anticipated.

**Conclusion:**

This study has applied a new method for analysing clinical data obtained from rehabilitation robotics studies. While the data obtained during the clinical trial is of multivariate nature, having multipoint and progressive nature, the multiple regression model used showed great potential for drawing conclusions from this study.

An important conclusion to draw from this paper is that this study has shown that the intervention and control phase both caused changes over a period of 9 sessions in comparison to the baseline. This might indicate that use of new challenging and motivational therapies can influence the outcome of therapies at a point when clinical changes are not expected.

Further work is required to investigate the effects arising from early intervention, longer exposure and intensity of the therapies. Finally, more function-oriented robot-mediated therapies or sling-suspension therapies are needed to clarify the effects resulting from each intervention for stroke recovery.

## Background

### Introduction

The GENTLE/S project was funded by the European Union under the Quality of Life initiative of Framework Five to evaluate robot-mediated therapy (RMT) in upper limb post stroke rehabilitation. Focusing on neurorehabilitation, one of the goals of the GENTLE/S project was to develop challenging and motivating therapies that would foster the patient's attention by means of level exercise interaction and the feeling of 'being in control' of their therapy session. GENTLE/S therapies are based on 'shaping' therapy, where the user can perform tailor made 'reach to a target' exercises in three dimensional space. This spatial configuration allows for the training of complex movements (for example, bringing an object close to the mouth or touching the forehead) mediated through the assistance of a sensorimotor, computer-based environment.

**Figure 1:**
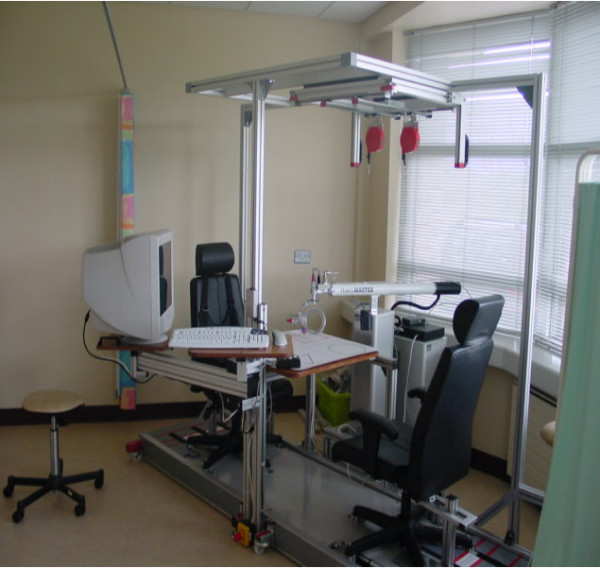
**The GENTLE/S system as used in the clinical trial**. The clinical prototype resulting from brainstorming with patients, clinicians, healthcare professionals and industrial parties.

**Figure 2:**
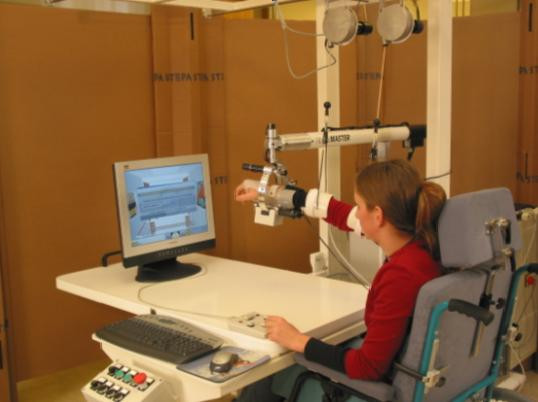
**Precursor commercial incarnation of GENTLE/S**. This figure depicts the controls for wheelchair docking, and controlling the arm support forces on the left. Patient controls are seen under the subject's left hand and a therapy can be chosen or halted and will only proceed if the 'operate' button is held down. The patient can 'eject' their arm from the HapticMaster.

Figure 1 illustrates the GENTLE/S system as used in the clinical trial while Figure 2 illustrates the precursor commercial incarnation of the system. The system comprises a 3 degree of freedom (DOF) robot manipulator (HapticMaster, FCS Robotics, the Netherlands) with an extra 3DOF passive gimbal mechanism, an exercise table, computer screen, overhead frame and chair. The 3DOF passive gimbal allows for pronation/supination of the elbow as well as flexion and extension of the wrist. A harness arrangement was built into the chairs to restrain the user's trunk movements. This could be used to achieve two desired effects. The first was to ensure that the patient would maintain a reasonably upright posture with only a limited ability to compensate using trunk movements. The second was that it was then possible to consider the shoulder as a fixed point and use this information to determine the pose of the user's arm. Exercise is delivered by the robot after the user's arm has been placed on an elbow orthosis suspended from the overhead frame and on the gimbal using a wrist splint. This arrangement of de-weighting the paretic arm was in part developed to minimise shoulder subluxation problems and also to compare with the control phase, sling suspension only. The exercise is executed only when an operation button is pressed by the user's unaffected arm or by the therapist.

The therapies that were programmed on the HapticMaster consisted of a series of reaching and withdrawing movements. The empirical minimum jerk approach [1] was used to pattern the reaching movement as it is simple to implement in real-time, and has some evidence that it represents at least the profile of human movements. The hypothesis suggests that human arm reaching movements tend to minimise the change of acceleration with respect to time (jerk) over the movement resulting in graceful and gentle movements [2]. This is normally expressed as a fifth or seventh order polynomial in a parametric time 0 <*t *<*duration *although changing the range to -1 <*t *< 1 simplifies the calculations. Thus equation EQ. 1 was used to derive the polynomial trajectory of an underlying preferred movement.

J=∫0d|d3x/dt3|2dt     EQ. 1
MathType@MTEF@5@5@+=feaafiart1ev1aaatCvAUfKttLearuWrP9MDH5MBPbIqV92AaeXatLxBI9gBaebbnrfifHhDYfgasaacH8akY=wiFfYdH8Gipec8Eeeu0xXdbba9frFj0=OqFfea0dXdd9vqai=hGuQ8kuc9pgc9s8qqaq=dirpe0xb9q8qiLsFr0=vr0=vr0dc8meaabaqaciaacaGaaeqabaqabeGadaaakeaacqWGkbGscqGH9aqpdaWdXbqaamaaemaabaGaemizaq2aaWbaaSqabeaacqaIZaWmaaGccqWG4baEcqGGVaWlcqWGKbazcqWG0baDdaahaaWcbeqaaiabiodaZaaaaOGaay5bSlaawIa7amaaCaaaleqabaGaeGOmaidaaaqaaiabicdaWaqaaiabdsgaKbqdcqGHRiI8aOGaemizaqMaemiDaqNaaCzcaiaaxMaacqqGfbqrcqqGrbqucqGGUaGlcqqGGaaicqaIXaqmaaa@495A@

**Figure 3:**
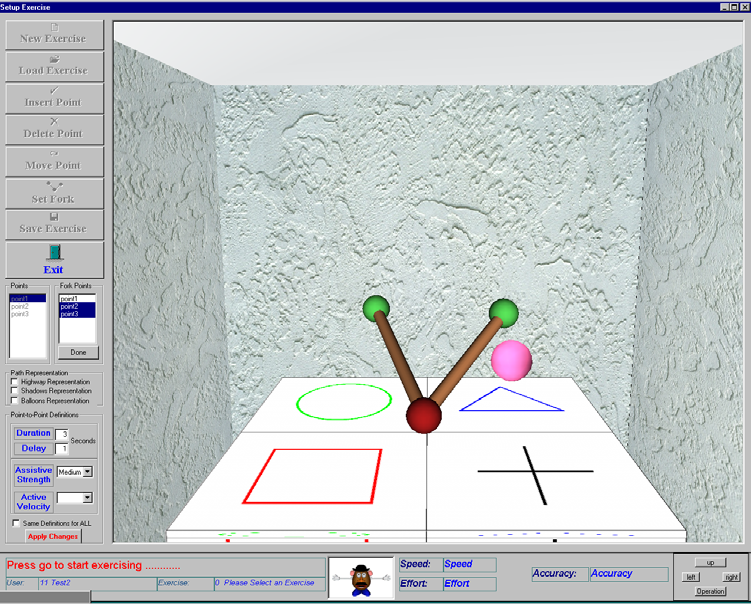
**The GUI used by the therapist in order to setup each exercise**. The GUI allows for easy setup of an exercise while moving the robot/patient arm to different positions in the workspace. Different points can be inserted or deleted and different levels of assistance can be chosen for each exercise.

**Figure 4:**
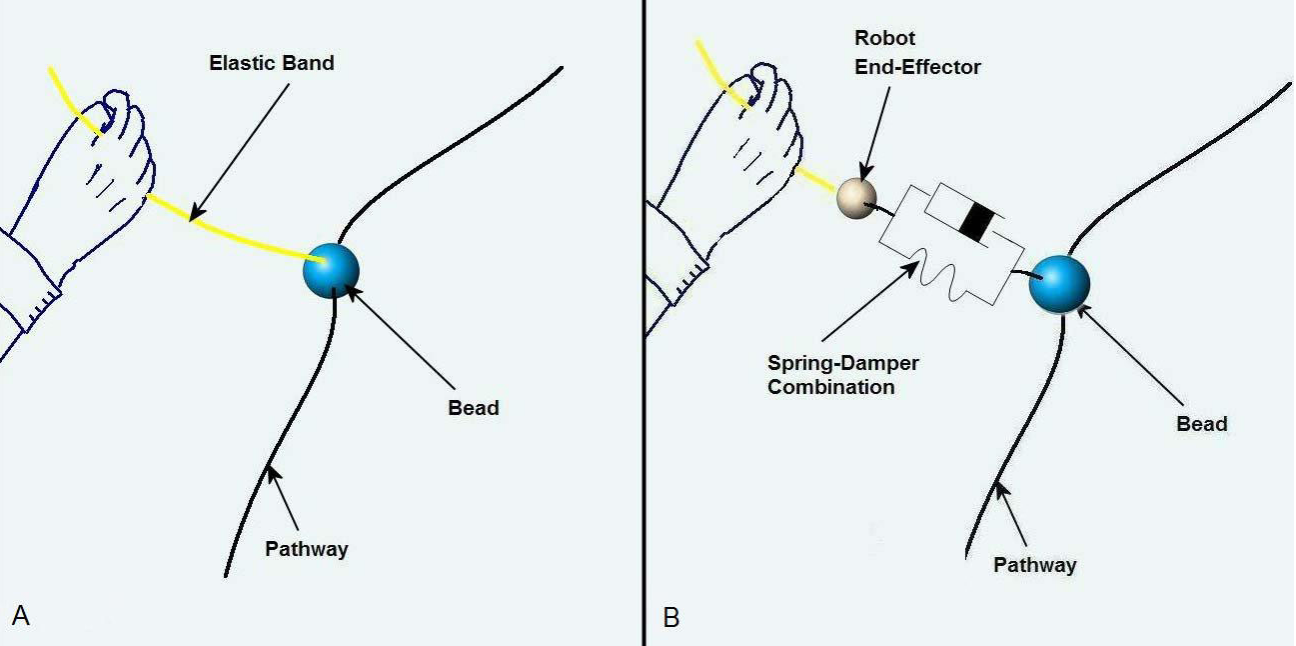
**The variable impedance concept**. A. The real life example of the bead-pathway concept. B. The bead-pathway concept implemented using spring-damper combination and pathway model using higher order polynomials.

The minimum jerk polynomial requires the therapist to define a start and end point and the duration of the movement. During the patient setup phase, a graphical user interface (GUI) is used to fine-tune a therapy session for each patient. The therapist can insert points in the workspace by moving the robotic arm to the desired starting and end points. Figure 3 shows the GUI used for customising the therapies to each patient. Multiple points could be inserted for one therapy session. Optionally the therapist can also define a maximum mid point velocity. The patient's own movement is encouraged to follow this trajectory by programming a variable impedance that is conceptually similar to attaching the patients hand using an elastic band to a bead placed on a flexible wire-path. This is termed as bead-pathway concept (Figure 4A). The therapist could also specify the strength of this conceptual elastic band. Figure 4B depicts the bead-pathway implementation using a spring-damper combination and the trajectory reproduced using the minimum jerk trajectory model.

**Figure 5:**
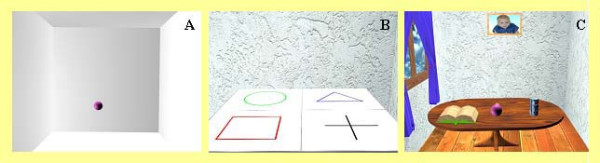
**The three difference virtual environments used for the trial**. A. Empty room – A simple environment that represents the haptic interface workspace and aims to provide early post-stroke subjects with awareness of physical space and movement. B. Real room – An environment that resembles what the patient sees on the table in the real world. The mat with 4 different shapes on the table (as seen in Figure 1) is represented in the 3D graphical environment. This environment was developed to help discriminating the third dimension that is represented on the Monitor 2D screen. C. Detail room – A high detail 3D environment of a room comprising of a table, several objects (a book, can of soft drink), portrait of a baby, window, curtains, etc.

**Figure 6:**
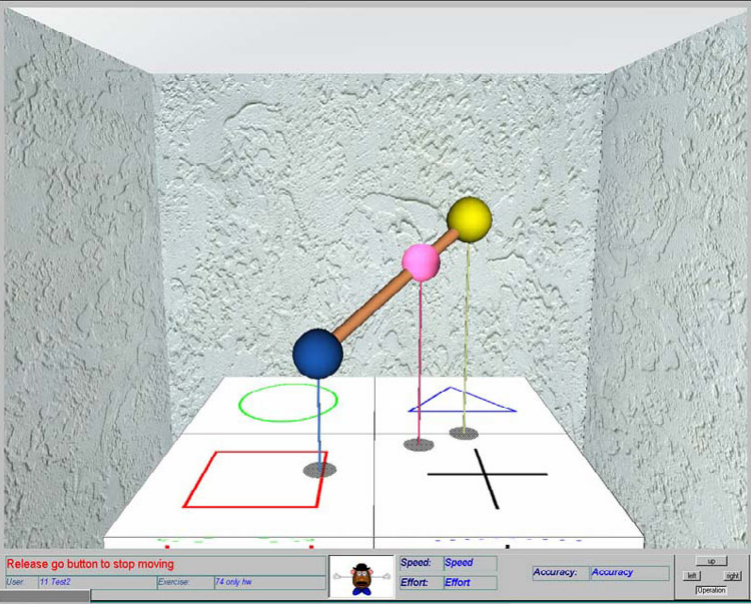
**An exercise setting during execution**. Subject's arm position is presented using the pink sphere. The start and end point of the trajectory are presented by the blue and yellow spheres. The start and end points are connected using a line providing guidance for execution. In addition to the table mat, the threads hanging from each sphere (termed as balloons threads) and the shadows are used to provide a better depth perception.

There is a selection of virtual environments which can be used as patients' workspace. Figure 5 shows some of these virtual rooms. Using the minimum jerk polynomials, a number of different therapy exercises were implemented on the prototype system. These therapies all use the selected virtual environment. During the therapy, the location of patient's arm is displayed on the screen using a pink sphere. Starting and end points of the movement are displayed using different colours. It is possible to have a guidance line connecting the starting point to the end point, providing a straight-line ruler for each task (Figure 6). Different therapeutic modes are implemented as described below.

### Patient Passive

The Patient Passive mode was the first therapy implemented and was intended for patients who have insufficient arm strength or neural connectivity to move. This is similar to therapies provided by existing machines and would simply stimulate sensory neurons. The primary difference is the virtual environment that is displayed where the patient is encouraged to observe the planned movement and think about how to make the movements. The HapticMaster moved the arm to follow the predefined path with the elastic band strength programmed by the therapist. When the patient's arm reaches the target, the movement would pause momentarily and then proceed to the next target point.

### Patient Active Assisted

For more capable patients the HapticMaster was programmed so that it would only start to move if the patient initiated a movement by providing a nominal force in the correct direction. This was done by comparing the force vectors recorded at the end-effector, to the position vector constituting the desired direction of the movement. A threshold value could be set during the setup phase to tune the sensitivity for movement initiation. After the initiation was made, the haptic interface assisted the user to reach to the end point again using bead-pathway concept.

### Patient Active

The third mode is the ratchet mode or the Patient Active mode. The user has an unlimited time to finish the task. This mode provides a unidirectional movement, where the amount of deviation can be controlled by changing spring-damper coefficients. Similar to the previous mode, the user initiates the right movement. The haptic interface stays passive until the user deviates from the predefined path. In this case, the spring-damper combination encourages the patient to return to the pathway. During this mode, the robot only assists the patient to correct deviations from the planned trajectory and the patient is solely responsible to reach from the start point to the end point defined. This operation will end on reaching the end point or releasing the operation button. Upon arrival at the end point, it is up to the user to continue the same movement back to the start point, a new point or end the whole session in this mode.

### Trajectory Fork

The trajectory fork was intended to augment other therapies and increase involvement in the activity by allowing the user to decide which movement to make. Before initiating a movement the user was presented with a set of alternate reaching goals and based on the initial forces exerted by the user on the HapticMaster, one of these goals would be selected and the trajectory calculated and initiated. From a clinical point of view, apart from providing the stroke patient with repetitive challenge therapies, the ability to choose was seen to increase the motivation and challenge of the therapy. It is notable that this mode was not used during the clinical trial and was only available on the precursor commercial model.

### Motivational Considerations

Various other methods were considered to increase the user's motivation and attention as these were seen as essential elements in the therapy to allow the brain to re-organise and adapt. The therapies were arranged to occur in a highly realistic 3D virtual environment and three were demonstrated in the precursor commercial prototype. These were, a simple room with a table, a set of supermarket shelves to allow reaching and selection of items from a shelf, and a home environment where items such as bottles could be selected. This was intended to be a staging point that would allow the user to eventually practice the actions needed to pour a drink. An additional activity was navigating through a simple maze game. Because the clinical trial was already in progress at the time when these considerations were made, none of the above rooms were present during the clinical trial. Other situations were also considered such as exploring a virtual museum and other games like activities.

The concept of giving performance cues following a therapy was considered but it was not possible to detail a sufficiently robust measure that could be used to score the success or otherwise of the movements.

### Objectives

The objective of the GENTLE/S study was to assess the effectiveness of the Robot-mediated therapies (RMT) compared to sling suspension (SS) therapies using a series of 31 single case studies conducted in two separate centres. This paper presents a new approach in analysing multivariate data obtained in clinical trial of the robotic system. This rational for using this new approach is the multivariate and progressive nature of the data and the complexity induced by the ABC-ACB clinical design. The next section describes the clinical trial and study design as used for the GENTLE/S project.

### Clinical Trial

The GENTLE/S clinical trial consisted of a series of 31 single case studies, using a randomised ABC-ACB design (ABC and ACB – explained further in the text). The centres involved in this trial were the Battle Hospital, Reading, United Kingdom and the Adelaide & Meath Hospital, Dublin, Republic of Ireland. Subjects at each centre were randomised into either ABC or ACB groups. Inpatient and outpatient participants were recruited by referral from their consultant. They were sought to be medically stable in order to cope with the duration of the trial. Participants were all following their first stroke and over 60 years of age with ability to give informed consent. In addition, they had to achieve a score higher than 24 in the Short Orientation Memory Concentration (SOMC) assessment. Participants with pacemakers were excluded from this study. The recruited patients attended three times per week for a period of nine weeks. They completed a baseline measurement phase (A, 8 measurements). It was in place to identify the current recovery status or baseline (BL). During this phase, no therapeutic intervention was provided. This was followed by a period of RMT (B, 9 measurements) and de-weighted sling suspension (C, 9 measurements). The order in which the B or C phase followed the baseline was decided based on subjects' randomisation into the A-B-C or A-C-B groups. Hence, the only difference between the two groups were the order in which the B or C phase were delivered. Since there is a suggested dose response to intervention [3], this design in the study permitted to control for the dose effects by allowing the comparison between different phases of the trial [4]. The demographic data of the subjects including gender, stroke paretic side, age and number of months post stroke are given in Table 1.

**Table 1:** Subject demographics for the GENTLE/S study

		Male	Female	Left Hemi	Right Hemi	Age	Post stroke
Reading (n = 11)	ABC Group (n = 6)	4	2	4	2	67 ± 6	19 ± 14.3
	ACB Group (n = 5)	4	1	3	2	67 ± 6	37.2 ± 19.5
	Subtotal	8	3	7	4	67 ± 6	27.2 ± 18.5

Dublin (n = 20)	ABC Group (n = 10)	3	7	5	5	66 ± 8	16 ± 9.4
	ACB Group (n = 10)	6	4	4	6	70 ± 11	25.6 ± 25
	Subtotal	9	11	9	11	68 ± 9	20.7 ± 19

Total		17	14	16	15	Years (Mean ± SD)	Months (Mean ± SD)

At the start of each trial session, for all three phases, subjects were assessed using validated outcome measures. These measures included the upper limb section of the Fugl-Meyer (FM), Motor Assessment Scale (MAS) and the active and passive goniometry for elbow and shoulder (G). Table 2 shows the randomisation used for the trial and the order of phase appearance based on this randomisation.

**Table 2:** Two randomised groups for the clinical trial

Weeks	1			2			3		4			5			6			7			8			9			
ABC Group	Baseline (Phase A)								Robot-Mediated Therapy (Phase B)									Sling Suspension (Phase C)									

Sessions	1	2	3	4	5	6	7	8	9	10	11	12	13	14	15	16	17	18	19	20	21	22	23	24	25	26	27

ACB Group	Baseline (Phase A)								Sling Suspension (Phase C)									Robot-mediated Therapy (Phase B)									

During the B phase, the subject received individually tailored robot-mediated therapy (RMT) using the GENTLE/S system. Three 10-minute sessions were conducted using one of the three therapy modes available (patient passive, patient active-assisted and patient active as mentioned earlier). Based on the patient's stroke severity and the type of support required, one of the above modes was chosen for each 10-minute session.

During the C phase, the subject's paretic arm was suspended from a frame eliminating gravity using sling suspension (SS) techniques. The subject was asked to use the de-weighted arm to perform different activities. Similar to the B phase, three 10-minute sessions were carried out during this phase. For the first section, the combined movement involving shoulder and elbow flexion and extension was exercised while patients lay on their side. The second 10-minute session required activities involving shoulder flexion and extension only, while the third 10-minute part involved elbow flexion and extension.

### The Fugl-Meyer outcome measure

The Fugl-Meyer (FM) scale is an impairment-based scale used to assess the motor deficits in neurological patients, mainly stroke survivors. It includes items of upper and lower-limb sensation and motor control. Listed items in this scale are scored between 0, 1, and 2 where a score of 2 denotes the ability to respond correctly to a listed item [5]. The scale consists of 62 items. Hence, the maximum score for the FM is 124 if the complete response given to all items is summed. This scale has previously been tested and shown to be both valid and reliable [6,7].

This scale is one of the most widely used instruments in clinical assessment [8]. Usually, the overall outcome of the instrument is calculated by summing the response given to each item or subscale, which can then be used in analytical models including statistical analysis [some examples in rehabilitation robotic literature include: [9-12]].

**Figure 7:**
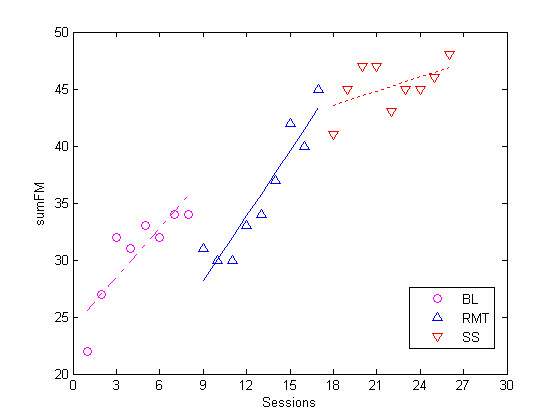
**Comparison between slopes of the regression line for different phases of the trial, one typical subject**. The sumFM scores from each phase is accompanied by a regression line calculated using the least square method.

One of the outcome measures used at the start of each session is the upper-limb section of this assessment. The GENTLE/S study concentrated only on treatment of the upper limb, thus only the upper-limb section of the FM (33 subscales) was chosen for this clinical study. The scores given to each subscale were summed to calculate the total score obtained in one session. Figure 7 presents the sums obtained during the clinical trial for one of the subjects at Battle Hospital, Reading. Linear regression was used to calculate the slope for each phase of the trial and the figure depicts these slopes. It can be seen that better recovery is achieved during the B phase where the slope is steeper. A MATLAB routine was used to calculate and automatically produce these figures at the end of each subject's trial period. However, due to the complex nature of the study design, in order to summarise the results statistically, a more advanced multiple regression model was used. The following sections will describe this model and analyse the results obtained from the clinical study.

## Methods

### Initial Analysis

**Figure 8:**
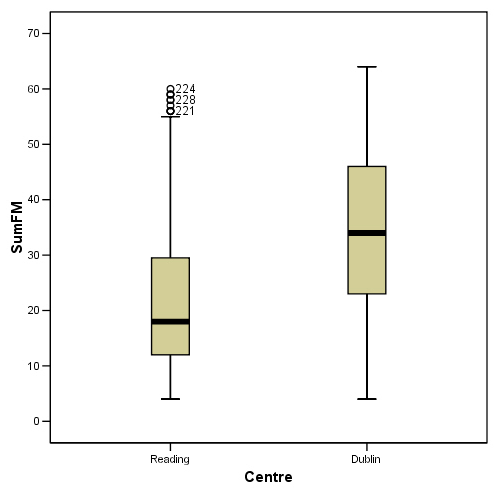
**Results Comparison between the two centres**. The differences in sumFM score is observed between the two centres involved.

As a first approach, the FM results were visually inspected using boxplots and case summaries. Figure 8 presents the boxplot comparing the results between the two centres involved. It depicts the differences observed between the two centres involved using the FM measure.

**Figure 9:**
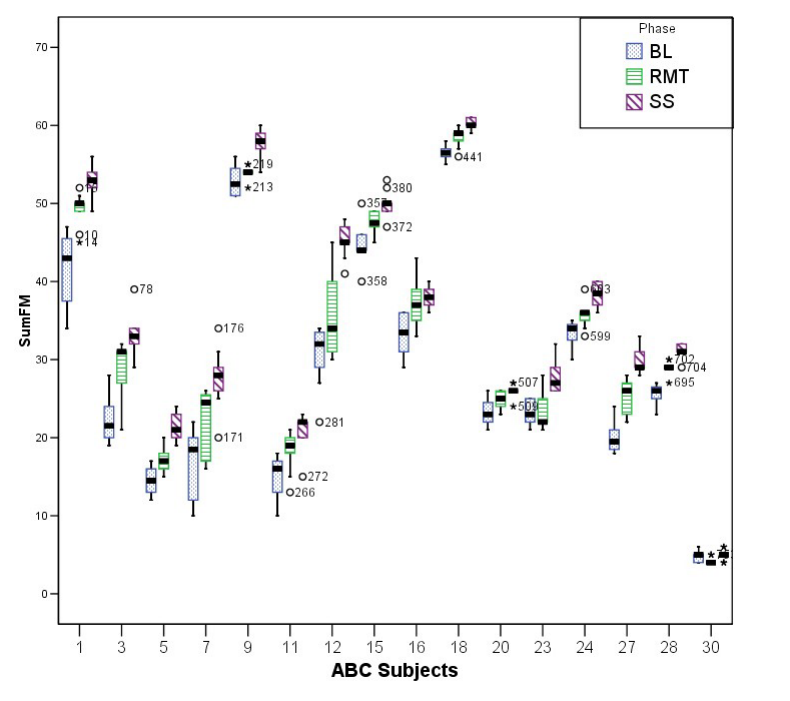
**The ABC group during the three phases of the trial**. Comparison between the three phases of the trial for the participants in the ABC group.

**Figure 10:**
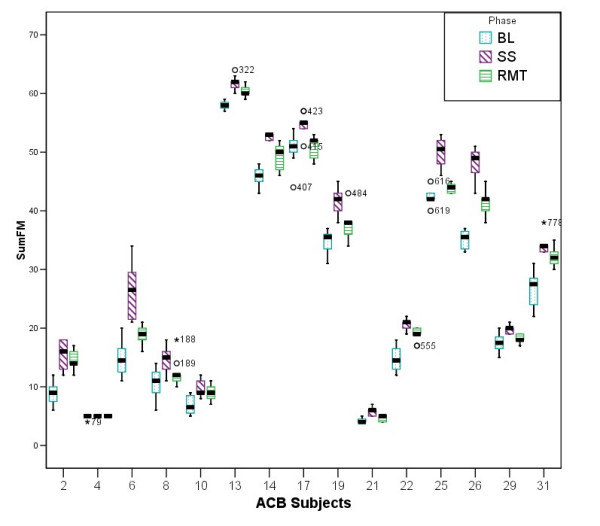
**The ACB group during the three phases of the trial**. Comparison between the three phases of the trial for the participants in the ACB group.

The boxplots shown in Figure 9 and Figure 10 illustrates the results obtained from comparing the three phases of the trial for subjects in ABC and ACB groups. The main objective was to identify any existing trend or any significant outlier in the data before proceeding with more thorough examination. In addition, these two figures show a general improvement trend when BL data is compared to the RMT or SS points. It is also noticeable that the SS results are generally better than the RMT results as depicted by their medians. On the other hand, RMT seems to have caused greater deviation in the scores measured (i.e compare subject 6 RMT phase to his/her SS phase)

### A multiple regression model

The next step was to use a general linear model (GLM) to identify different parameters contributing to the variance seen in the recorded trends. The GLM is an advanced form of ANOVA allowing analysis of multiple levels of unbalanced data. This was chosen because during clinical studies, it was not always possible to obtain a balanced design as subjects may have missed a therapy session due to ill health or other causes. The GLM used '*centre*', '*grouping*', '*subject*', and '*session*' as its model parameters. The results showed strong and statistically significant effects for all these parameters indicating the difference between different centres, different groupings (ABC and ACB), and inherent differences between different subjects. It also showed that the performance between different sessions had been diverse demonstrating a positive or negative trend or change during the trial. Knowing such differences, it could be possible to continue the analysis in each centre and group separately but this would have resulted in reducing the number of data points and hence, losing statistical power. A better and more advanced model was needed to analyse the data without breaking it into fragments.

Noting that one of the objectives of the study was to compare subjects' progress during the different phases of the trial, a multiple regression model was reasonable. Figure 7 illustrates how this approach might work with a straight line being fitted to each of the trial phases. Using the least squares linear regression method provides the slope and intercept as well as fit statistics for each subject. Moreover, it is possible to devise a similar technique to analyse the total trend for all subjects by considering more independent parameters (such as centre, grouping and subjects) in this formulation.

Multiple regression is a common way to assess co-variations between and among different variables [13]. It can be used to consider multiple independent variables when calculating the least square estimates for a complex data set. Using a multiple regression analysis, we can devise our model using the EQ. 2:

y=bc.centre+bBL.BL+bRMT.RMT+bSS.SS+∑i=1n−1bsi.Subjecti+c+e     EQ. 2
 MathType@MTEF@5@5@+=feaafiart1ev1aaatCvAUfKttLearuWrP9MDH5MBPbIqV92AaeXatLxBI9gBaebbnrfifHhDYfgasaacH8akY=wiFfYdH8Gipec8Eeeu0xXdbba9frFj0=OqFfea0dXdd9vqai=hGuQ8kuc9pgc9s8qqaq=dirpe0xb9q8qiLsFr0=vr0=vr0dc8meaabaqaciaacaGaaeqabaqabeGadaaakeaacqWG5bqEcqGH9aqpcqWGIbGydaWgaaWcbaGaem4yamgabeaakiabc6caUiabdogaJjabdwgaLjabd6gaUjabdsha0jabdkhaYjabdwgaLjabgUcaRiabdkgaInaaBaaaleaacqWGcbGqcqWGmbataeqaaOGaeiOla4IaemOqaiKaemitaWKaey4kaSIaemOyai2aaSbaaSqaaiabdkfasjabd2eanjabdsfaubqabaGccqGGUaGlcqWGsbGucqWGnbqtcqWGubavcqGHRaWkcqWGIbGydaWgaaWcbaGaem4uamLaem4uamfabeaakiabc6caUiabdofatjabdofatjabgUcaRmaaqahabaGaemOyaiMaem4Cam3aaSbaaSqaaiabdMgaPbqabaGccqGGUaGlcqWGtbWucqWG1bqDcqWGIbGycqWGQbGAcqWGLbqzcqWGJbWycqWG0baDdaWgaaWcbaGaemyAaKgabeaakiabgUcaRiabdogaJjabgUcaRiabdwgaLbWcbaGaemyAaKMaeyypa0JaeGymaedabaGaemOBa4MaeyOeI0IaeGymaedaniabggHiLdGccaWLjaGaaCzcaiabbweafjabbgfarjabc6caUiabbccaGiabikdaYaaa@7973@

where *b*_*c *_represents the coefficient for the centre parameter. As there were only two centres involved in this study, only one binary variable is needed in the model. The BL, RMT and SS slopes represented by a *b *variable subscripted with the correct label, considers the slope for each phase of the trial accompanied by the sessions attended in each phase. Subjects form completely independent categories and pose independent effects on this model. To represent this variable with more than two categories, dummy coding or indicator coding is required. Dummy coding is a way of including nominal or ordinal variables in a regression equation. Each independent category except one is added as a dichotomy. The omitted variable provides a baseline for comparison while avoiding multicollinearity. Each subject is represented by an independent subject variable and its slope represented by *bs*_*i *_with a subscript indicating the variable index. Only one subject is excluded from this coding and hence is the range of the subscript (*n - 1*). The penultimate coefficient, *c*, is the intercept for the regression line and *e *presents the modelling error.

The SPSS statistical package was used to analyse the fitness of the above model. The variable calculated for the sum of Fugl-Meyer (*sumFM*) outcome in each session was used as the dependent variable. For the independent variables, additional preparation was needed. To analyse this model using SPSS, data was stored in the form of rows representing every measurement session for each subject (8 BL measures, 9 RMT measures, 9 SS measures) and columns representing each variable in the above model. As an example, the *S*_2 _variable, which represents the second subject in this regression model, is represented by one column in the datasheet. It is set to 1 for all rows relating to this subject and 0 for all other rows that represent other subjects. Other subjects were also inserted using a similar approach. This technique allows for establishing subject independence in the model. Subject's data is represented by a value of one in *S*_2 _column while other subjects have no effect on this variable due to having zero values. It is notable that this technique allows for reducing one of the variables because a column with all zero values can represent one of the subjects as a baseline subject. Hence the number of dummy variables is usually one less than the number of categorical variables. SPSS is capable of detecting multicollinearity and excluding those variables causing multicollinearity. A more in-depth explanation on using and creating these variables is given in 'regression with dummy variables' [14].

One objective of this model was to compare the effectiveness of our control (SS) to our intervention (RMT) and to the baseline (BL) phase. The BL, RMT and SS columns show the session numbers as a sequential number assigned to each session. The BL column was filled with session (ordinal) numbers (values ranging from 1 to 8) during this phase. The rest of cases for this column were equal to zero indicating that no two phases happened simultaneously. The RMT and SS variables were inserted similarly. A question arises regarding grouping effects and whether there would be any effect arising from differences between the ABC and ACB, that are to be considered in this model. Having a separate phase indicator (BL, RMT and SS) will take into account the grouping effects; recalling from Table 2, the BL, RMT and SS have their sessions numbered sequentially so that, if the RMT is presented before SS, or after it, it will have session numbers varying between 10–18, or 19–27 consequently. This will automatically include the grouping parameter into the model. The multiple regression model was then executed to identify the coefficients and their statistical significance. The next two sections further explain the multiple regression parameter method used and the cross-validation procedure employed. The reader with less statistical interest can continue with the 'Analysis of the Results'.

### Parameters 'forced entry'

The multiple regression model designed and implemented with the previous parameters was entered into the SPSS linear regression analysis. The 'Enter' method was used which forces the model to consider all variables as significant variables in the model (see Table 3). In addition, Table 4 shows the coefficients calculated for each variable entered into the model.

**Table 3:** Multiple Regression Model Summary

Model	R	R Square	Adjusted R Square	Std. Error of the Estimate	Change Statistics				
					
					R Square Change	F Change	df1	df2	Sig. F Change
1	.988	.975	.974	2.660	.975	898.792	33	754	.000

**Table 4:** Multiple Regression, model Coefficients

Model	Unstandardized Coefficients		Standardized Coefficients	t	Sig.	95% Confidence Interval for B	
1	B	Std. Error	Beta			Lower Bound	Upper Bound

(Constant)	.932	.644		1.449	.148	-.331	2.196
Baseline	.114	.053	.021	2.144	.032	.010	.219
**RMT**	**.207**	**.018**	**.117**	**11.635**	**.000**	**.172**	**.241**
**SS**	**.324**	**.017**	**.187**	**18.639**	**.000**	**.290**	**.358**
Subject1	43.230	.763	.467	56.684	.000	41.733	44.728
Subject2	8.634	.762	.093	11.337	.000	7.139	10.129
Subject3	23.461	.763	.254	30.762	.000	21.964	24.958
Subject5	13.000	.763	.141	17.045	.000	11.502	14.497
Subject6	15.350	.769	.163	19.966	.000	13.841	16.860
Subject7	17.353	.770	.184	22.536	.000	15.841	18.864
Subject8	8.057	.762	.087	10.579	.000	6.562	9.552
Subject9	50.038	.763	.541	65.610	.000	48.541	51.535
Subject10	4.065	.769	.043	5.288	.000	2.556	5.574
Subject11	13.230	.763	.143	17.348	.000	11.733	14.728
Subject12	32.976	.765	.356	43.102	.000	31.474	34.478
Subject13	55.632	.785	.567	70.854	.000	54.091	57.173
Subject14	44.788	.762	.484	58.807	.000	43.293	46.283
Subject15	42.805	.771	.454	55.535	.000	41.292	44.318
Subject16	31.526	.763	.341	41.297	.000	30.027	33.025
Subject17	47.738	.762	.516	62.622	.000	46.241	49.234
Subject18	53.976	.765	.583	70.551	.000	52.474	55.478
Subject19	33.956	.764	.367	44.449	.000	32.457	35.456
Subject20	19.884	.763	.215	26.072	.000	18.387	21.381
Subject21	.584	.762	.006	.766	.444	-.913	2.080
Subject22	14.149	.764	.153	18.521	.000	12.649	15.648
Subject23	20.257	.763	.219	26.535	.000	18.758	21.756
Subject24	31.431	.772	.333	40.706	.000	29.915	32.947
Subject25	41.314	.794	.412	52.011	.000	39.755	42.874
Subject26	37.078	.769	.393	48.233	.000	35.569	38.587
Subject27	20.449	.763	.221	26.787	.000	18.951	21.948
Subject28	23.807	.763	.257	31.216	.000	22.310	25.305
Subject29	14.610	.780	.152	18.740	.000	13.079	16.140
Subject30	.168	.765	.002	.220	.826	-1.334	1.670
Subject31	26.596	.762	.287	34.920	.000	25.101	28.091

Note that subject 4 was selected as the baseline subject. The selection of this subject was mainly due to presence of minute variations in the subject score during all phases of the trial. As this study sought to investigate progress, comparing other subjects to this subject would provide a reasonable base of comparison while avoiding multicollinearity and inclusion of unnecessary variables.

### Cross-validation of the model

The linear regression model in Equation 1 assumes errors have a normal distribution. Thus after fitting, the residual errors can be compared to a normal distribution as an aid towards cross-validation using SPSS. Histograms of the residuals and the normal probability plots were produced for the standardised residuals. These suggested that the assumption of the error distribution is reasonable.

The next step was to investigate the statistical power. The method used by Dunlap et al. was used to calculate the statistical power [15]. For the alpha-level (0.05) and the sample size used (*n *= 788), the program used the population correlation coefficient obtained from the model summary table to calculate the statistical power (*p *= 1.0). This was in agreement with the power value suggested by the G*Power program developed and presented by Erdfelder et al. [16].

Another validation method used was data splitting. Using SPSS, 60% of the data was randomly selected to estimate coefficients for a new model. Table 5 presents the model summary produced for this regression:

**Table 5:** Multiple Regression Model Summary for the Random 60% of the Data

Model	R	R Square	Adjusted R Square	Std. Error of the Estimate	Change Statistics				
					
					R Square Change	F Change	df1	df2	Sig. F Change
2	. 987	.975	.973	2.694	.975	511.097	33	435	.000

The final step was to cross-validate the adjusted R^2 ^using Stein's formula as seen in EQ.3 [See [17], page 118]:

AdjustedR2=1−[(n−1n−k−1)(n−2n−k−2)(n+1n)](1−R)2     EQ. 3
 MathType@MTEF@5@5@+=feaafiart1ev1aaatCvAUfKttLearuWrP9MDH5MBPbIqV92AaeXatLxBI9gBaebbnrfifHhDYfgasaacH8akY=wiFfYdH8Gipec8Eeeu0xXdbba9frFj0=OqFfea0dXdd9vqai=hGuQ8kuc9pgc9s8qqaq=dirpe0xb9q8qiLsFr0=vr0=vr0dc8meaabaqaciaacaGaaeqabaqabeGadaaakeaacqWGbbqqcqWGKbazcqWGQbGAcqWG1bqDcqWGZbWCcqWG0baDcqWGLbqzcqWGKbazcqWGsbGudaahaaWcbeqaaiabikdaYaaakiabg2da9iabigdaXiabgkHiTmaadmaabaWaaeWaaeaadaWcaaqaaiabd6gaUjabgkHiTiabigdaXaqaaiabd6gaUjabgkHiTiabdUgaRjabgkHiTiabigdaXaaaaiaawIcacaGLPaaadaqadaqaamaalaaabaGaemOBa4MaeyOeI0IaeGOmaidabaGaemOBa4MaeyOeI0Iaem4AaSMaeyOeI0IaeGOmaidaaaGaayjkaiaawMcaamaabmaabaWaaSaaaeaacqWGUbGBcqGHRaWkcqaIXaqmaeaacqWGUbGBaaaacaGLOaGaayzkaaaacaGLBbGaayzxaaWaaeWaaeaacqaIXaqmcqGHsislcqWGsbGuaiaawIcacaGLPaaadaahaaWcbeqaaiabikdaYaaakiaaxMaacaWLjaGaeeyrauKaeeyuaeLaeiOla4IaeeiiaaIaeG4mamdaaa@656B@

Where *n *is the total number of cases, *k *is the number of predictors and *R*^2 ^is the unadjusted value obtained from the model summary. This resulted in the value of 0.972, which is very close to the value calculated by SPSS and presented in Table 3.

## Analysis of the results

Table 3 and Table 4 show the results as calculated for the statistical model. The multiple R is a gauge of how well the model predicts the observed value and is presented by the R column in the summary table (Table 3). A value of 1 indicates a situation where the model perfectly predicts its observed values. The value of 0.988 given in this table presents this model as a good predictor of the observed values. The R^2 ^value shows the amount of variation in the outcome, which is accounted for by the model. The value of 0.975 indicates that 97.5% of the variation seen in the outcome is accounted for by the model. The next important output in this table is the adjusted R^2^. It gives some idea of how well this model generalises. Ideally this should be close to the observed R^2^. These results show good agreement between these two values. The adjusted R^2 ^value cross-validated using the Stein's formula is 0.972, which is in agreement with both values mentioned previously. The next column presents the standard error of the estimated values. A small standard error indicates that most sample means from the estimated values are similar to the population mean and so the estimated values are likely to be an accurate presentation of the population.

The next important section of the results is the F-Change statistics. The F-ratio is a measure of how much a model has improved the prediction of the outcome compared to the level of inaccuracy of the model [18]. For these data, *F *is 898.792, which is significant at *p *< 0.001. This model causes R^2 ^to change from zero to 0.975 and this change in the amount of variance explained gives rise to an F-ratio of 898.792. This change in F-ratio indicates improvement in prediction due to the model and the statistically significant p-value indicates that there is less than 0.1% chance that an F-ratio of this size would occur by chance alone. It can be concluded that the regression model results in a significantly better prediction than if we used mean values of scored FM results for each trial session and each subject. In other words, the regression model is a better choice for tracking progression in subjects' scores compared with the calculated mean values.

Having established the model using its summary table, Table 4 presents the model coefficients. These are the parameters calculated for the equation 1. These values indicate the individual contribution of each predictor in the model. Replacing b-values given by B column in this table will provide the regression equation for the GENTLE/S results. Our main objective in this study was to compare the effects caused by the RMT to those observed from the SS phase of the trial. These results are found in the two highlighted (bold typeface) rows of Table 4. The b-value calculated for the RMT phase is 0.207 indicating that for the RMT session, the FM score changes by 0.207 units. The b-values calculated for the SS phase is 0.324, indicating that better improvements in the FM score can be attributed to this exercise. As there were nine sessions in each phase of the trial, from these results we can conclude that the SS phase has advanced the FM scores by 1.053 (9 × (0.324 - 0.207)) over RMT. This indicates a modest and small change in the FM score due to the SS phase compared to the RMT phase. The standard error associated with each b-value indicates the extent that these values would vary across different samples of the population.

Another important section of this table is the t-statistics. The t-test indicates whether each b-value differs significantly from zero, in other words, whether each predictor is making a significant contribution to the model. Both coefficients (RMT and SS) show significant p-values for this test indicating their contribution to the model with statistical significance. The larger the value of t, the greater is the contribution to the model. This also shows that the SS improves the *sumFM *score more than the RMT, although the extent of this contribution is modest [19]. Another important observation is exclusion of the centre parameter. SPSS application is able to exclude un-necessary variables to avoid multicollinearity. Table 4 does not present this variable and detailed output from the model (not presented here) shows that centre variable was partially correlated with other parameters involved in the model (subject variable), thus failing the multicollinearity test.

A final statement from these results can be drawn from the standardised Beta, which shows the number of standard deviations that the outcome will change as a result of one standard deviation change in each predictor. In scenarios where indicators have different standard deviations and different units, the b-values using the unit change in the score due to unit change in the indicator do not provide a good basis for comparison while the standardised Beta is formulated in terms of unit change of standard deviation and provides a better ground for comparison. The standard deviations calculated for the RMT and SS phase indicators are 9.363 and 9.574 respectively. The standard deviation for the FM score is 16.536. The RMT Beta indicates that 9.363 change in the RMT would result in 1.93 (16.536 × 0.117) change in the FM score. The SS Beta column indicates that 9.574 change in the SS would result in 3.09 (16.536 × 0.187) change in the FM score. Hence, based on the standardised Beta values, the SS phase causes the FM score to change 1.16 unit more compared to the RMT phase. This is similar to the results calculated from b-values because the RMT and SS indicators have similar and close standard deviations in our case.

**Figure 11:**
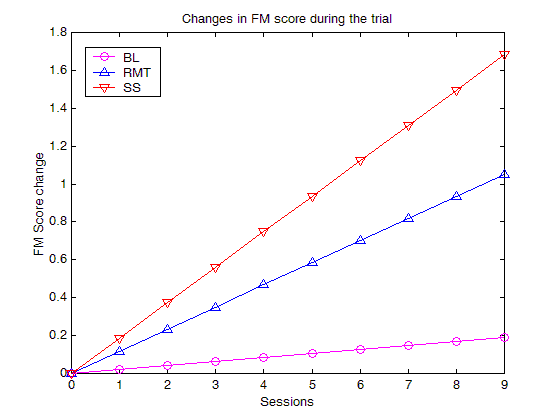
**Comparison between progress during each phase of the trial**. The slopes calculated by the statistical models is plotted to compare between different phases of the trial. Arbitrary session numbers is used to allow for this comparison.

Based on the standardised Beta values, Figure 11 presents a comparison plot evaluating the difference between the baseline, RMT and SS phases.

## Discussion

The highly multivariate nature of clinical trials of rehabilitation accompanied by a recovery trend for individual subjects calls for the use of advanced analytical approaches. It is important to mention that different statistical models can be used to analyse a selected dataset and our choice in this paper is only examining one possible model for this analysis. In this paper, we have used a multivariate model to draw inference on the data from a small study (*n *= 31). The technique has shown great potential in analysing multipoint multi-variable progressive clinical data and should be used more widely, but care is needed to ensure that the model is not overfitted. The cross validation effort presented here is an important part of the procedure made to ensure a best fit for multivariate models.

The presented model is capable of summarising data at different levels, i.e. centre, phase and subject and of identifying the influential factors that affect the recovery trend in a group of subjects undergoing clinical trial. It also provides the potential to forecast the outcome of individual subjects in a near future, providing a chance for active feedback during therapy period. Using this model and the coefficients calculated, one can see that longer exposure to both interventions could influence the recovery more. However, an extension of this research can investigate the dose-response and response to intensity of RMT or SS compared to the GENTLE/S trial.

The model used provided a first insight to the results obtained from trialling the GENTLE/S robotic system. It provided a chance to compare different indicators used in the model in terms of their contributions to the total variance seen in the data. In spite of differences between the two centres involved, the model showed that variations were caused mainly by different subjects attending the trial and the phasing of the trial. The centre indicator was eliminated due to its colinearity with other parameters in the model. However, it is notable that the issue of inter/intra rater reliability was not sought during the trial and also that the therapists involved in each centre were aware of the objectives of the study as well as subjects' randomisation. Noting these, the model still provided a chance to summarise the data by empowering individual subjects as different independent variables. It is noteworthy that this paper only presented the results obtained from analysing the FM outcome measures and further publications aim to investigate the remaining outcomes as collected during the GENTLE/s clinical trial.

The main objective of this study was to compare between the RMT phase of the trial relative to the other two phases. Both phases showed improvement relative to the BL phase as can be seen in Figure 11. It is important to recall that subjects involved in this study only received 30 minutes of each intervention for each session. However, the changes shown are statistically significant many months after stroke providing evidence for recovery when no clinical changes are anticipated. Although statistics showed that the RMT phase was slightly less effective than the SS phase, the extent of this difference is clinically disregarded (within the given time period). On the contrary, it is notable that accepted clinical measures may not have the sensitivity required for this comparison while robotics and advanced instrumentation techniques have the potential to provide quantitative scores of arm function. A study of the joint angle versus torque plots presented by Mak et al. showed that it is possible to identify the direction of energy flow between human and robot. This could inform the recovery process if one is measuring subject's contribution to an assisted task [20].

It is worth mentioning that both RMT and SS phases helped the arm against gravity by de-weighting it. It is possible to argue that perhaps similar results between the two phases could be entirely due to using similar suspension systems. Recent research by Sukal et al. has investigated the abnormal torque patterns in hemiparetic limbs and its response to supported and unsupported movements. They have shown that supported movement have the potential to influence paretic arm's reaching envelope. Their research has also shown the potential of robotic and anti-gravity supports in quantifying muscle coordination after stroke [21]. The results shown by this paper regarding the SS intervention also showed that by de-weighting the arm against gravity and practicing different groups of arm movements, subjects involved in this clinical trial showed better results compared to the intervention tested and also compared to the baseline phase where no therapy was administered. This was achieved in spite of only 30 minutes of suspension therapies. It can be suggested that future research can use de-weighting in conjunction with longer and more intense therapy, performance feedback and a motivating therapeutic context in order to investigate the usefulness of arm suspension more thoroughly. This can have further use for home-based rehabilitation systems where subjects are allowed to use a system within their own private home.

The results draws conclusion from a total of 4.5 hours RMT interventions per subject and a small number of subjects. This is not comparable to other clinical studies, ie drug trials. Larger number of subjects and longer exposure to both therapies, in addition to higher resolution assessment techniques are seen as important factors required for comparison between the control and intervention phases. A point to consider in future studies is the lack of balance between different groups within each centre and between the two centres. A more balanced design in addition to double-blinding procedure allows for more accurate conclusions. Insertion of a baseline phase after each intervention, ie ABACA-ACABA study design can also enable the researchers to investigate the direct effects caused by an intervention, ie C phase in ABC, without need to worry about carry over effects caused by interjecting B before C.

During the trial, it was observed that each subject's therapy was changed over time and did not provide a common exercise, which could be compared to previous attempts. To use more accurate measures such as power flow in clinical trials, it is necessary to have both repeatable and varying elements present during a therapy session. The repeatable exercises would allow identifying the changes observed while varying components in each exercise would make the therapy more exciting.

All GENTLE/S therapies were targeting the arm movement but disregarded grasping. However, the main objective of many arm movements is to grasp and manipulate the environment. It can be argued that absence of grasping made the therapies less exciting and less realistic resulting in less improvement than expected. In addition, interactive content leading to making decisions are usually present in daily living activities. Future research should focus on more directed reaching, grasping and decision making to make the interaction more realistic.

## Conclusion

One of the objectives of the GENTLE/S study was to investigate the effects arising from the RMT in comparing it to the SS and the BL phase. The methodology used here shows competence in facilitating this comparison. This paper showed that multiple regression can be used to investigate the differences between diverse variables more thoroughly. Although the results presented in this paper show small differences between these RMT and SS phases, subject's exposure to these phases was not long and the therapy sessions were not intense, happening only 3 times a week, and were well beyond the normal period when recovery is normally expected. It is important to mention that some patients did not achieve a stable baseline during this phase. Ideally, these observations are continued until a stable baseline is achieved. However, due to timelines imposed by the funded study, continuing the baseline phase was not possible. Further studies should consider more flexible timelines to allow for such observations.

The methodology used here has showed differences between the two interventions involved, RMT and SS, to the level of one point on the FM scale, while the FM scale itself lacks the resolution for this type of comparisons. It can be suggested that the methodology itself is capable of detecting small changes in similar studies. Future studies can benefit from biomechanical measures that offer better resolution in conjunction with the clinical outcomes.

This study has applied a new method for analysing clinical data obtained from rehabilitation robotics studies. While the data obtained during the clinical trial is of multivariate nature, having multipoint and progressive nature, the multiple regression model used showed great potential for drawing conclusions from this study. This approach allows for investigating the effect of different indicators' contribution into total score variations. These indicators included phase, centre and subject. The results showed that the variations in both centres involved are insignificant in comparison to the effects caused by the SS or RMT interventions as well as inherent differences existing between different subjects.

A final conclusion to draw from this paper is that this study has shown that RMT and SS both caused changes over a period of 9 sessions in comparison to the baseline. This might indicate that use of new challenging and motivational therapies can influence the outcome of therapies at a point when clinical changes are not expected. Future studies are needed to investigate effects resulting from motivational context and interactive functional content as well as feedback during therapies. Such therapies can take place using the robot-mediated therapy or the sling suspension of the arm. However, the virtual reality and feedback mechanism is also a likely promoter to recovery and can be a target for future investigations. Further work is required to investigate the effects arising from early intervention, longer exposure and intensity of the therapies. Finally, more function-oriented RMT or SS therapies are needed to clarify the effects resulting from each intervention for stroke recovery.

## Competing interests

The author(s) declare that they have no competing interests.

## Authors' contributions

FA conceived of this methodological study. He was involved in multivariate model design and execution, statistical analysis and coordination activities. He was also responsible for drafting this manuscript. RL provided feedback throughout this methodological study and assisted with drafting the manuscript. EG was one of the research physiotherapists who conducted the clinical trial, which included the Fugl Meyer measurements and collected the data from the Reading patients. CC was responsible for the clinical trial in Reading and also assisted in the design of the clinical study. WH was the coordinator of the GENTLE/S project and provided advice and feedback on the manuscript. GJ was also a leading partner during the GENTLE/S project and has contributed to detailed discussions on the methodology as well as providing help with the manuscript.
